# Clinical value of thromboelastography in predicting the risk of recurrence of acute ischemic stroke

**DOI:** 10.3389/fneur.2024.1420915

**Published:** 2024-08-16

**Authors:** Ruyue Guo, Xiaoming Shen, Jin Lu, Jiao Zhou, Shinan Hao, Rui Lan, Yumin Xu

**Affiliations:** ^1^Department of Encephalopathy, the First Affiliated Hospital of Henan University of Traditional Chinese Medicine, Zhengzhou, China; ^2^The First Clinical Medical College of Henan University of Traditional Chinese Medicine, Zhengzhou, China

**Keywords:** thromboelastography, recurrent stroke, influencing factors analysis, predictive value, acute ischemic stroke

## Abstract

**Background:**

Thromboelastography (TEG) can objectively reflect the formation, development and rupture process of thrombosis in patients, but there are limited data on whether TEG can be used as a predictive tool for recurrence in patients with acute ischemic stroke.

**Objective:**

To explore the TEG risk of recurrence in patients with acute ischemic stroke predictive value.

**Methods:**

A total of 441 patients with acute ischemic stroke who met the research criteria in the First Affiliated Hospital of Henan University of Traditional Chinese Medicine from January 2020 to December 2021 were selected as the research objects. TEG was measured in all patients, and the main parameters of TEG (R value, indicating coagulation reaction time; K value and Angle, the rate of blood clot formation; MA value, indicating the maximum amplitude). The primary outcome of this study was ischemic stroke recurrence. Recurrent events included cerebral infarction, cerebral hemorrhage, TIA, and were determined by combining imaging events and clinical events. Logistic regression analysis was used to explore the influencing factors of recurrence in patients with acute ischemic stroke.

**Results:**

Fifty-six patients (12.7%) had recurrence. Multivariate Logistic regression analysis showed that: Age [OR = 1.078, 95%CI(1.024, 1.135)], triglyceride [OR = 1.541, 95%CI(1.033, 2.298)], glycosylated hemoglobin [OR = 1.401, 95%CI(1.097, 1.790)], history of hypertension [OR = 16.046, *p* < 0.05], 95%CI(4.726, 54.489), R value [OR = 0.533, 95%CI(0.351, 0.809)], MA value [OR = 1.399, 95%CI(1.004, 1.949)] were independent influencing factors for hemorrhagic transformation in patients with acute ischemic stroke.

**Conclusion:**

TEG has some value in predicting recurrence in patients with acute ischemic stroke, and the MA value in TEG [AUC = 0.806 (95%CI:0.747–0.867), with a sensitivity of 78.6% and a specificity of 70.4%], predicted the most significant efficiency of AIS recurrence.

## 1 Introduction

Acute ischemic stroke (AIS) is one of the most common cerebrovascular diseases worldwide, and it is also one of the most important causes of disability and death worldwide, which has caused a significant burden on individual health, family and society (1). According to the World Health Organization, about 15 million people suffer from stroke every year, about one third of them will die, and another third will suffer severe disability (2). AIS has a high recurrence rate, and studies have shown that about a quarter of patients experience recurrence within 5 years after initial stroke (3). Despite significant progress in the acute management and preventive measures of AIS in recent years, the risk of AIS recurrence remains high, and the mortality and disability rates after recurrence are significantly increased. Therefore, effective prediction of the recurrence of AIS is essential to implement preventive measures and improve patient outcomes.

Numerous scientific studies have established that the recurrence of ischemic stroke is associated with risk factors such as age, gender, hypertension, diabetes, coronary heart disease, family history, unhealthy lifestyle habits, serum protein levels, total cholesterol, high-density lipoprotein cholesterol, and coagulation function (4–6). These studies have formed a solid foundation for predicting the risk of recurrent ischemic stroke. Currently in clinical practice, various scales are primarily used to predict the risk of recurrent ischemic stroke; examples include the Essen scale (7) and SP-IIscale (8). Despite achieving some degree of effectiveness in predicting the risk of recurrent ischemic stroke based on current domestic and international research status using these scales and predictive factors adopted thus far; most variables utilized in these studies are predominantly traditional indicators such as personal history and past records. As a result their predictive accuracy is relatively low and they are unable to explain or verify potential relationships between variables. In recent years with advancements in medical technology however thromboelastography (TEG) has seen increasing clinical application across multiple fields including surgery, liver disease cardiovascular disease, and trauma. This demonstrates its potential value in predicting and managing patients’ coagulation function (9). The unique feature of TEG lies in its ability to comprehensively assess a patient’s coagulation status including parameters such as coagulation time, clot formation speed, clot strength, and fibrinolysis etc. (10), which holds particular significance for stroke patients given that their coagulation function is related to recurrence risks (11). Traditional coagulation indicators like PT, TI, and APTT fail to effectively reflect overall clotting function or thrombus strength information. Moreover they are easily influenced by anticoagulant drugs thereby limiting their predictive value for thrombus formation and prognosis after strokes (12). Despite demonstrating potential applications within these areas, TGE’s role efficacy remains insufficiently researched when it comes to predicting AIS recurrences.

Therefore, the aim of this study was to explore the potential value and application of TEG in predicting AIS recurrence. By detecting TEG in AIS patients and tracking their recurrence, we hope to identify the TEG parameters associated with the risk of recurrence, and then provide more personalized risk assessment and prevention strategies for AIS patients.

## 2 Materials and methods

### 2.1 General data

The case data in this study were derived from 441 AIS patients who were treated in the First Affiliated Hospital of Henan University of Traditional Chinese Medicine from January 2020 to December 2021. All the enrolled patients had undergone TEG measurement. Inclusion criteria: ① Conform to the guidelines of diagnosis and treatment of acute ischemic stroke in China 2018 (13) midbrain stroke related diagnostic criteria; ② Diagnosis of acute ischemic stroke according to medical history and CT/MRI; ③ no hepatitis B, hepatitis C, AIDS, syphilis and other infectious diseases; ④ age ≥ 18 years old; Patients or their legal guardians provided written informed consent. Exclusion criteria: ① Transient ischemic attack (TIA), hemorrhagic stroke and mixed stroke diagnosed by western medicine; ② Ischemic stroke diagnosed by western medicine but with disturbance of consciousness (including coma, lethargy, drowsiness, confusion and delirium), TCM diagnosis belongs to the viscera of stroke; ③ Severe liver and kidney failure, malignant tumor, gastrointestinal bleeding; ④ unable to cooperate with clinical data collection; ⑤ pregnant and lactating women; ⑥ patients with serious missing clinical data (data missing rate > 20%). TEG and related examinations were performed after admission, and the patients were followed up by outpatient examination, telephone and other methods for 1 year. Patients with recurrence were enrolled as observation group, and patients without recurrence were enrolled as control group. The follow-up time was 1 year. The study was approved by the ethics committee of the investigator’s hospital (approval number: 2022HL-088-01).

### 2.2 Methods

This study was a prospective clinical registry study. According to literature research and expert consultation in the early stage of the project, we developed a clinical data collection table. The relevant data of the selected patients included: General demographic data (age, gender), personal history (continuous smoking history, continuous drinking history, exercise), condition at onset (BMI index, systolic blood pressure at admission, diastolic blood pressure at admission), past history (TIA, hypertension, diabetes, coronary heart disease, hyperlipidemia, hyperhomocysteinemia), laboratory biochemical indicators (red blood cell count, white blood cell count) number, platelet count, hemoglobin concentration, total cholesterol, triglyceride, low density lipoprotein, high density lipoprotein, apolipoprotein A1, apolipoprotein B, plasma prothrombin time, fibrinogen content, activated partial thromboplastin time, thrombin time measurement, glycosylated hemoglobin, creatinine, uric acid, homocysteine, TOAST classification (large artery porgrage) Arteriosclerosis type, arteriole type (including lacunar), cardiac origin, other causes), related scales (mRS Total score, NIHSS total score).

After AIS patients were admitted to hospital, the median cubital vein blood was taken by the nurses in the Department of encephalopathy, placed in the sodium citrate anticoagulant tube, and sent to the test within 2 h. The test was performed by the laboratory physician in the thrombelastography laboratory of our hospital. The test process is fully automated. The specific parameters of TEG in patients: reaction time (R value), coagulation time (K value), coagulation Angle (Angle), maximum amplitude of thrombus (MA value) level.

The general demographic data, personal history, conditions at onset, past history, laboratory biochemical indexes, TOAST classification, NIHSS scale score, mRS Scale score and main parameters of TEG were compared between patients with and without recurrence after stroke, and the influencing factors of recurrence in patients with acute ischemic stroke were analyzed.

### 2.3 Study variables

#### 2.3.1 Outcome indicators

The outcome indicators in this study included primary outcome indicators and secondary outcome indicators. The primary outcome was recurrent events during the ischemic stroke follow-up period, and the secondary outcome was neurological deterioration.

##### 2.3.1.1 Primary outcome measure

Recurrent events include cerebral infarction, cerebral hemorrhage, and TIA (14, 15). The determination of recurrent events should be based on reliable imaging indicators and doctors’ clinical evaluation, combined with imaging events and clinical events.

##### 2.3.1.2 Secondary outcome measure

Neurological function deterioration was demonstrated by experts according to CHANCE criteria (16).

#### 2.3.2 Time of measurement

The study used three times of on-site follow-up and five times of telephone. On-site follow-up was required for each patient at 12, 24, and 48 weeks after enrollment, and telephone follow-up at 8, 20, 28, 36, and 44 weeks and at the end of follow-up. Predictors of relapse were measured and recorded at baseline enrollment and on-site follow-up. Outcomes are captured and recorded during follow-up and need to be supported by objective and valid clinical evidence (imaging reports or medical records).

#### 2.3.3 Outcome evaluation

The end event evaluation expert group is composed of one group leader and four members, a total of five people. The evaluation method is as follows: The detailed clinical records and image data of recurrent ischemic stroke events are sorted out, and the information of the patient and the receiving doctor (including the information reflected in the image examination and physical and chemical examination) is hidden, and the final judgment and evaluation is made by the expert group of the end event.

### 2.4 Statistical methods

In the study, SPSS 25.0 statistical software was used for univariate analysis of general data, including t test or Mann–Whitney *U* test for quantitative data and two test for qualitative data. In this study, *p* < 0.05 was taken as the criterion to indicate a statistically significant difference.

## 3 Results

### 3.1 General situation

The results of this study showed that a total of 441 AIS patients were included. According to the recurrence within 1 year after discharge, the subjects were divided into two groups, including 56 cases in the recurrence group and 385 cases in the non-recurrence group, with a recurrence rate of 13.7%. The oldest patient was 88 years old and the youngest was 33 years old, with an average age of 63.97. There were 263 males (59.6%) and 178 females (40.3%).

### 3.2 Comparison of baseline data (general demographic data, past history, laboratory biochemical indexes, TOAST classification, exercise or not, mRS Scale score, NIHSS scale score) between recurrence and non-recurrence patients

The age, BMI index, history of hypertension and hyperhomocysteinemia in recurrent AIS patients were higher than those in non-recurrent AIS patients. The proportion of male patients with recurrence was higher than that of female patients. The proportion of non-recurrence in patients who adhered to exercise was higher than that in recurrent AIS patients. The proportion of recurrent patients with small arterial occlusive according to TOAST classification was higher than that of non-recurrent patients; The results showed that age, BMI index, history of hypertension, history of hyperhomocysteinemia, exercise, triglyceride, activated partial thromboplastin time, glycated hemoglobin A1c, TOAST classification of small artery occlusion were the risk factors of recurrence in AIS patients (*p* < 0.05).If AIS patients relapse factors: Gender, systolic blood pressure at onset, diastolic blood pressure at onset, TIA history, hyperlipidemia history, coronary heart disease history, continuous smoking history, continuous drinking history, NIHSS score, mRS Score, red blood cell count, white blood cell count, platelet count, hemoglobin concentration, total cholesterol, low density lipoprotein, high density lipoprotein, apolipoprotein A1, apolipoprotein B, plasma Prothrombin time, thrombin time, fibrinogen content determination, creatinine, uric acid, homocysteine, TOAST classification for the proportion of cardiac and other reasons, there was no statistically significant difference(*p* > 0.05), as presented in Table 1.

**Table 1:** The baseline data (general demographic data, past history, laboratory biochemical indexes, TOAST classification, exercise or not, mRS Scale score, NIHSS scale score) of patients with recurrence and without recurrence were compared.

Variables of interest	Entry items	No recurrence group	The recurrence group	Test statistic values	*p*
Age	M(Q1, Q3)	64(55,71)	66(64.25,71.75)	−2.261^a^	0.024^*^
Gender					
Female	n(%)	161(41.8)	17(30.3)	2.668	0.102
Male	n(%)	224(58.1)	39(69.6)		
BMI index	M(Q1, Q3)	23.88(22.49,25.86)	24.58(23.67,27.66)	−2.430^a^	0.015^*^
Blood pressure at the time of onset					
Systolic blood pressure at the time of onset	M(Q1, Q3)	149(135,160)	150(135,160)	−0.719^a^	0.475
Diastolic blood pressure at onset	M(Q1, Q3)	90(80,96)	87.5(78.5,93.75)	−1.363^a^	0.173
Previous medical history					
History ofTIA	n(%)	34(8.8)	6(10.1)	0.210	0.647
History of diabetes mellitus	n(%)	295(76.6)	35(62.5)	5.178	0.023^*^
History of hypertension	n(%)	166(43.1)	52(92.8)	48.388	0.000^*^
History of hyperlipidemia	n(%)	35(9.0)	8(14.2)	1.499	0.221
Coronary heart disease	n(%)	65(16.8)	13(23.2)	1.346	0.246
Hyperhomocysteinemia	n(%)	5(1.2)	4(7.1)	8.352	0.004^*^
Previous history of smoking and drinking					
History of continuous smoking	n(%)	92(23.8)	17(30.3)	1.097	0.295
History of continuous drinking	n(%)	44(11.4)	9(16.0)	0.997	0.318
Exercise or not	n(%)	144(37.4)	13(23.2)	4.293	0.038^*^
Scale score					
NIHSS score	M(Q1, Q3)	3(2,6)	4(2,7)	−0.328^a^	0.743
mRS Score	M(Q1, Q3)	1(1,2)	1(1,2.75)	−0.929^a^	0.353
Laboratory biochemical indicators					
Red blood cell count (10^12^/L)	M(Q1, Q3)	4.49(4.20,4.79)	4.53(4.22,4.83)	−0.649^a^	0.517
White blood cell count (10^9^/L)	M(Q1, Q3)	6.71(5.19,7.80)	6.66(5.49,7.52)	−0.268^a^	0.789
Platelet count (10^9^/L)	M(Q1, Q3)	218(180,255.5)	206(164.25,269.25)	−0.806^a^	0.420
Hemoglobin concentration (g/L)	M(Q1, Q3)	137(128,147.5)	138(129.25,147)	−0.523^a^	0.601
Total cholesterol (mmol/L)	M(Q1, Q3)	4.58(3.74,5.40)	4.59(3.61,5.23)	−0.614^a^	0.539
Triglyceride (mmol/L)	M(Q1, Q3)	1.40(1.03,1.99)	1.55(1.22,4.01)	−3.102^a^	0.002^*^
Low density lipoprotein (mmol/L)	M(Q1, Q3)	2.64(2.09,3.26)	2.68(2.05,3.24)	−0.122^a^	0.903
High density lipoprotein (mmol/L)	M(Q1, Q3)	1.16(1.00,1.35)	1.09(0.93,1.28)	−1.637^a^	0.102
Apolipoprotein A1 (g/L)	M(Q1, Q3)	1.20(1.08,1.32)	1.20(1.01,1.28)	−0.681^a^	0.496
Apolipoprotein B (g/L)	M(Q1, Q3)	0.91(0.72,1.10)	0.94(0.75,1.10)	−0.241^a^	0.809
Plasma prothrombin time (s)	M(Q1, Q3)	11.8(10.8,12.5)	11.69(10.23,12.3)	−1.167^a^	0.243
Fibrinogen content (g/L)	M(Q1, Q3)	2.88(2.37,3.47)	3.04(2.63,3.58)	−1.634^a^	0.102
Activated partial thrombin time (s)	M(Q1, Q3)	29.2(26.4,31.0)	31.05(28.0,33.18)	−1.959^a^	0.050^*^
Thrombin Time Measurement (s)	M(Q1, Q3)	16.1(15.0,16.8)	15.9(15.1,16.8)	−0.492^a^	0.623
Glycosylated hemoglobin	M(Q1, Q3)	6.09(5.40,6.90)	7.88(6.07,9.52)	−4.590^a^	<0.001^*^
Creatinine (umol/L)	M(Q1, Q3)	59.0(50.0,71.0)	60.1(52.1,72.7)	−0.750^a^	0.453
Uric acid (umol/L)	M(Q1, Q3)	284(235,341)	280(229.25,316.25)	−1.161^a^	0.245
Homocysteine (umol/L)	M(Q1, Q3)	13.0(9.27,17.97)	13.99(9.85,18.23)	0.434^a^	0.664
TOAST classification					
Large artery atherosclerosis (LAA)	n(%)	275(71.4)	4(7.1)	9.786	0.002^*^
Small artery occlusion type(SA)	n(%)	104(28.5)	51(91.0)	10.438	0.001^*^
Cardiac origin (CE)	n(%)	4(1.0)	0(0.0)	0.587	0.444
Other Reasons (SOE)	n(%)	2(0.5)	1(1.7)	1.160	0.281

a represents the Z value, and the rest of the test statistic represents the χ^2^value. BMI, Body Mass Index, TOAST classification, etiological classification of stroke. *Indicates that the difference was statistically significant (*p* < 0.05).

### 3.3 Comparison of TEG parameters between patients with and without recurrence

The R value of recurrent AIS patients was shorter than that of non-recurrent AIS patients, and the Angle Angle and MA value of recurrent AIS patients were higher than those of non-recurrent AIS patients, and the differences were statistically significant (*p* < 0.05). There was no significant difference in *K* value between recurrent and non-recurrent AIS patients (*p* > 0.05), as shown in Table 2.

**Table 2:** Comparison of TEG parameters between patients with and without recurrence.

Variables of interest	Entry items	No recurrence group	The recurrence group	Test statistic values	*p*
*R*(min)	M(Q1, Q3)	6.2(5.3,7.2)	4.8(4.2,5.3)	−7.485^a^	<0.001
*K*(min)	M(Q1, Q3)	1.8(1.5,2.2)	1.75(1.5,2.0)	−1.394^a^	0.163
Angle(°)	M(Q1, Q3)	63.7(59.4,67.5)	69.9(66.6,73.7)	−7.226^a^	<0.001
MA (mm)	$\bar{x}\pm s$	57.84 ± 6.29	64.98 ± 5.36	−8.082^b^	<0.001

a represents the Z value.

b represents the t value.

The R value represents the coagulation reaction time, the K value and the Angle Angle represent the rate of blood clot formation, and the MA value represents the maximum amplitude.

### 3.4 Univariate and multivariate logistic analysis of influencing factors of recurrence in AIS patients

With the presence or absence of AIS recurrence as the dependent variable (assignment: Yes =1, no =0) The variables with statistical differences in Tables 1, 2 were independent variables, and univariate Logistic regression analysis was performed. The results showed that age, triglyceride, glycosylated hemoglobin, history of diabetes, history of hypertension, history of hyperhomocysteinemia, exercise, TOAST classification of large artery atherosclerosis type and small artery occlusion type, *R* value, Angle Angle, MA value may be the influencing factors of AIS recurrence (*p* < 0.05), see Table 3.

**Table 3:** Univariate logistic regression analysis of the influencing factors of recurrence in AIS patients.

	Assignment of value	*B*	SE	Wald *χ^2^*	*p*	Exp(B)
Age	Measured value	0.037	0.015	5.772	0.016	1.038
BMI index	Measured value	0.077	0.047	2.661	0.103	1.080
Triglycerides	Measured value	0.709	0.126	31.674	<0.001	2.031
Activated partial thromboplastin time	Measured value	0.056	0.032	3.057	0.080	1.057
Glycosylated hemoglobin	Measured value	0.336	0.061	29.921	<0.001	1.399
Diabetes	Yes =1, no =0	−0.676	0.301	5.044	0.025	0.508
Hypertension	Yes =1, no =0	2.842	0.529	28.865	<0.001	17.151
Hyperhomocysteinemia	Yes =1, no =0	1.766	0.687	6.608	0.010	5.846
Exercise or not	Yes =1, no =0	−0.681	0.334	4.171	0.041	0.506
Large artery atherosclerosis	Yes =1, no =0	−1.571	0.531	8.739	0.003	0.208
Small arterial type (lacunar)	Yes =1, no =0	1.406	0.482	8.510	0.004	4.080
Of cardiac origin	Yes =1, no =0	−19.285	20096.485	0.000	0.999	0.000
Other Reasons	Yes =1, no =0	1.248	1.233	1.023	0.312	3.482
Value of R	Measured value	−0.920	0.141	42.331	<0.001	0.399
Angle of view	Measured value	0.210	0.032	42.284	<0.001	1.234
Value of MA	Measured value	0.220	0.032	48.684	<0.001	1.246

The recurrence of AIS patients was taken as the dependent variable, and the variables with statistical differences in the univariate Logistic regression analysis were taken as independent variables to conduct multivariate Logistic regression analysis.The results showed that age, triglyceride, glycosylated hemoglobin, hypertension history, R value and MA value were independent risk factors for relapse in AIS patients (*p* < 0.05), as shown in Table 4.

**Table 4:** Multivariate logistic regression analysis of factors influencing recurrence in AIS patients.

	*B*	SE	Wald *χ^2^*	*p*	*OR*	95%CI	
Age	0.075	0.026	8.326	0.004	1.078	1.024	1.135
Triglycerides	0.432	0.204	4.496	0.034	1.541	1.033	2.298
Glycosylated hemoglobin	0.337	0.125	7.283	0.007	1.401	1.097	1.790
Diabetes	0.825	0.581	2.017	0.156	2.281	0.731	7.121
Hypertension	2.775	0.624	19.800	<0.001	16.046	4.726	54.489
Hyperhomocysteinemia	2.119	1.840	1.327	0.249	8.326	0.226	306.685
Exercise or not	−0.525	0.478	1.210	0.271	0.591	0.232	1.508
Large artery atherosclerosis	−1.681	1.663	1.022	0.312	0.186	0.007	4.845
Small arterial type (lacunar)	0.401	1.553	0.067	0.796	1.493	0.071	31.338
Value of R	−0.629	0.213	8.751	0.003	0.533	0.351	0.809
Angle of view	−0.132	0.170	0.602	0.438	0.876	0.628	1.223
Value of MA	0.336	0.169	3.940	0.047	1.399	1.004	1.949
Constant quantity	−21.079	4.688	20.213	0.000	0.000		

### 3.5 Evaluation of the clinical validity of the predictors

**Figure 1:**
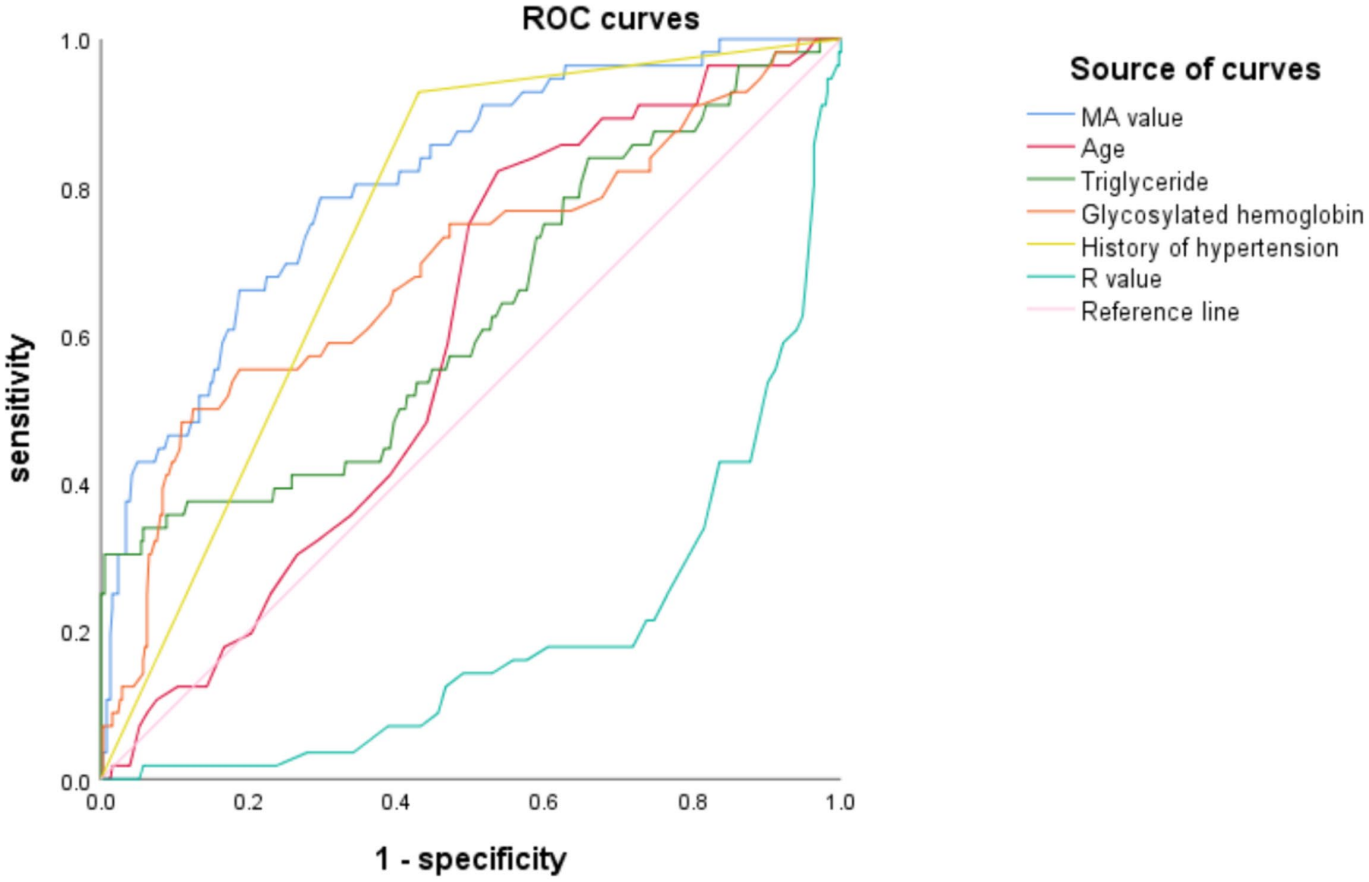
ROC curves of independent risk factors.

The receiver operating characteristic (ROC) curves were used to evaluate the independent risk factors of AIS recurrence as follows: Based on the multivariate Logistic regression analysis, the ROC curve analysis results of age, triglycerides, HbA1c, hypertension history, *R* value, and MA value showed *p* < 0.05, indicating that the above 5 independent risk factors have a significant ability to predict AIS recurrence. The AUC of MA value was 0.806 (95%CI: 0.747–0.867), the AUC of hypertension history was 0.749 (95%CI: 0.693–0.806), and the AUC of HbA1c was 0.690 (95%CI, 0.607–0.772) were the top three, indicating that the above three independent risk factors have a more significant ability to predict AIS recurrence. The results are shown in Table 5 and Figure 1.

**Table 5:** Area below the ROC curve for independent risk factors.

Test the outcome variable	Region	Standard error^a^	The asymptotic significance^b^	Asymptotic 95% confidence interval	
				Lower limit	Superior limit
Glycerin trilaurate	0.629	0.043	0.002	0.544	0.714
Glycosylated hemoglobin	0.690	0.042	0.000	0.607	0.772
Hypertension	0.749	0.029	0.000	0.693	0.806
R price	0.191	0.031	0.000	0.131	0.251
MA price	0.807	0.031	0.000	0.747	0.867

To test the outcome variables triglycerides, HbA 1 c, hypertension, R values, MA values with at least one binding value between the positive actual state group and the negative actual state group. The statistics may be biased.

a Assume as non-parametric.

b Null hypothesis: true region = 0.5.

The detection results show that the MA value has the most significant ability to predict AIS recurrence, and the critical value for predicting AIS with the MA value is 61.25, with a sensitivity of 78.6% and a specificity of 70.4%.

## 4 Discussion

Acute ischemic stroke (AIS) is a common neurological disease, and its etiology is related to the formation of atherosclerotic plaques, hemodynamic abnormalities, and coagulation/anticoagulation disorders. Its recurrence rate is relatively high, which seriously affects the quality of life and prognosis of patients (17). Abnormal coagulation function detected by conventional coagulation tests is associated with an increased risk of recurrent stroke (18). MAINO et al. found that increased thrombophilia was significantly associated with the risk of recurrent ischemic stroke (19). TEG can continuously, dynamically and quantitatively record the process of blood clot formation, comprehensively reflect the interaction of coagulation factors, platelets and FIB, and has important value in predicting bleeding tendency and thrombosis in patients (20). Studies have shown that TEG parameters are closely related to the coagulation status of patients with ischemic stroke in the acute phase, and may be associated with long-term prognosis (21).

Our findings are consistent with previous studies, which showed that specific TEG parameters, especially the maximum thrombus intensity (MA), were independent risk factors for AIS recurrence. MA mainly evaluates when blood clots reach their maximum intensity, which increases hypercoagulability and reduces platelet dysfunction, thrombocytopenia, or hypofibrinogenemia (22). Through TEG testing in 44 patients with acute ischemic stroke treated with thrombolysis, some scholars found that MA was associated with poor clinical score, that is, the lower the MA score with the progress of anticoagulation therapy, the better the clinical prognosis of patients (23).The positive association of MA and the risk of cardiovascular and cerebrovascular events was also reported in the study by Shi et al. (24). While our study strengthened the clinical relevance of this finding with a larger sample size and a longer follow-up period, further confirming the effectiveness of using TEG as a more dynamic and comprehensive assessment tool. Furthermore, the use of TEG may also help identify high-risk patient populations that are not detectable by conventional coagulation tests, providing a basis for personalized preventive treatment. For example, for patients with high thrombosis tendency indicated by TEG markers, physicians may choose a more aggressive anticoagulation strategy. This is in line with the study by Paciaroni et al., who found that a personalized anticoagulation regimen was effective in reducing the risk of recurrence in stroke patients (25).

However, there are some limitations to our study. First, as an observational study, we could not identify a direct link between TEG parameters and stroke recurrence from causality. Second, despite our relatively large sample size, the study remained limited to a single center, possibly limiting the general applicability of the results. Future studies will need to be conducted in multiple centers to validate our findings.

In conclusion, this study confirmed the potential value of TEG parameters, especially MA values, in predicting the risk of recurrent ischemic stroke. These findings provide a scientific basis for the clinical use of TEG as a risk assessment and management tool for patients with ischemic stroke. In the future, on the basis of previous studies, relevant imaging omics data and biomarker information of AIS patients should be further collected, and more studies on ischemic lesions should be conducted, such as studies on the identification of ischemic penumbral band in AIS patients by OEF map generated by quantitative magnetic resonance susceptibility mapping (QSM) (26). Further explore the application of TEG in different stroke subtypes, while considering individual patient differences, and build a recurrence risk assessment model based on machine learning to reduce the recurrence risk of ischemic stroke patients, and ultimately improve the prognosis and quality of life of patients.

## Data availability statement

The original contributions presented in the study are included in the article/supplementary material, further inquiries can be directed to the corresponding author.

## Ethics statement

The studies involving humans were approved by Ethics Committee of the First Affiliated Hospital of Henan University of Traditional Chinese Medicine. The studies were conducted in accordance with the local legislation and institutional requirements. The participants provided their written informed consent to participate in this study.

## Author contributions

RG: Data curation, Formal analysis, Investigation, Methodology, Writing – original draft. XS: Conceptualization, Supervision, Validation, Writing – review & editing. JL: Data curation, Writing – original draft. JZ: Data curation, Investigation, Writing – original draft. SH: Data curation, Investigation, Writing – original draft. RL: Supervision, Writing – review & editing. YX: Project administration, Writing – review & editing.
